# Characterization of experimental diabetic neuropathy using multicontrast magnetic resonance neurography at ultra high field strength

**DOI:** 10.1038/s41598-020-64585-1

**Published:** 2020-05-05

**Authors:** Daniel Schwarz, Asa S. Hidmark, Volker Sturm, Manuel Fischer, David Milford, Ingrid Hausser, Felix Sahm, Michael O. Breckwoldt, Nitin Agarwal, Rohini Kuner, Martin Bendszus, Peter P. Nawroth, Sabine Heiland, Thomas Fleming

**Affiliations:** 1 Department of Neuroradiology, Heidelberg University Hospital, INF 400, Heidelberg, Germany; 2 Department of Medicine I and Clinical Chemistry, Heidelberg University Hospital, INF 410, Heidelberg, Germany; 3 Institute of Pathology IPH, Heidelberg University Hospital, INF 224, Heidelberg, Germany; 4 Department of Neuropathology, Institute of Pathology, University Hospital Heidelberg, INF 224, Heidelberg, Germany; 5 CCU Neuropathology, German Consortium for Translational Cancer Research (DKTK), German Cancer Research Center (DKFZ), Heidelberg, Germany; 6 Pharmacology Institute, Medical Faculty Heidelberg, Heidelberg University, INF 366, Heidelberg, Germany; 7 German Center for Diabetes Research (DZD), Helmholtz Center Munich, Neuherberg, Germany; 8 Joint Division Molecular Metabolic Control, German Cancer Research Center (DKFZ), Heidelberg Center for Molecular Biology (ZMBH) and Heidelberg University Hospital University, Heidelberg, Germany; 9 Institute for Diabetes and Cancer IDC Helmholtz Center Munich and Joint Heidelberg-IDC Translational Diabetes Program, Neuherberg, Germany

**Keywords:** Magnetic resonance imaging, Experimental models of disease

## Abstract

In light of the limited treatment options of diabetic polyneuropathy (DPN) available, suitable animal models are essential to investigate pathophysiological mechanisms and to identify potential therapeutic targets. *In vivo* evaluation with current techniques, however, often provides only restricted information about disease evolution. In the study of patients with DPN, magnetic resonance neurography (MRN) has been introduced as an innovative diagnostic tool detecting characteristic lesions within peripheral nerves. We developed a novel multicontrast ultra high field MRN strategy to examine major peripheral nerve segments in diabetic mice non-invasively. It was first validated in a cross-platform approach on human nerve tissue and then applied to the popular streptozotocin(STZ)-induced mouse model of DPN. In the absence of gross morphologic alterations, a distinct MR-signature within the sciatic nerve was observed mirroring subtle changes of the nerves’ fibre composition and ultrastructure, potentially indicating early re-arrangements of DPN. Interestingly, these signal alterations differed from previously reported typical nerve lesions of patients with DPN. The capacity of our approach to non-invasively assess sciatic nerve tissue structure and function within a given mouse model provides a powerful tool for direct translational comparison to human disease hallmarks not only in diabetes but also in other peripheral neuropathic conditions.

## Introduction

*In vivo* magnetic resonance neurography (MRN) of the peripheral nervous system (PNS) has recently identified distinct focal lesions within the sciatic nerve as a major hallmark of diabetic polyneuropathy (DPN) in both type 1 (T1D) and type 2 diabetic (T2D) patients^1–3^. Such focal lesions have been reported to have a proximal predominance at thigh level and correlate closely to the degree of clinical severity.

In addition, these lesions have been shown to exhibit well-defined signal features in MRN: Quantification of either the signal in T2-weighted (T2-w) imaging or proton spin density (PD) indicated an increase within such lesions, suggestive of an increase of free-water molecules, as it would occur in endoneurial oedema, and a change in the organization of the extracellular compartment^1,2,4^. Recently, it was also found that in T2D hypointense nerve alterations can occur on T2-w imaging with fat saturation, suggesting an intraneurial deposition of lipids^3^. Furthermore, the measurement of fractional anisotropy (FA), which reflects the spatial anisotropy in the extent of water molecule self-diffusion, was reduced within DPN nerve lesions, whereas apparent diffusion coefficient (ADC), which reflects the average extent of water molecule self-diffusion, was increased. The changes observed with respect to FA and ADC^5,6^ would again be suggestive of a change within the macromolecular compartment consistent with a loss of axons and myelin.

A direct correlation between peripheral nerve imaging and the pathological nature of the lesions from patients with DPN has been difficult to achieve, as the fascicle biopsies from such proximal sites are not possible. However, this is not a limiting factor in experimental models of DPN. One of the most widely used animal models is the use of streptozotocin (STZ) to induce a phenotype which mimics T1D in mice. The presence of hyper- and hypoalgesic phenotypes as well as electrophysiological alterations have traditionally been interpreted as consequence of murine DPN^7^. Intraepidermal nerve fibre densities (IENFD) in skin-punch biopsies have shown that nerve fiber loss is a characteristic feature in experimental DPN^8–10^. However, evidence remains limited as to the degree to which changes in the proximal nerve, as have been reported in the analysis of patients with DPN^11,12^, play a role in such models^13,14^.

The objective of this study is therefore to implement a novel multicontrast, ultra high field MRN approach to assess pathomorphological nerve changes at a very high resolution which can be applied to mice *in vivo*. Such a non-invasive imaging toolbox may allow for detection of potential nerve lesions and distinct MR-signatures as correlate of experimental DPN and other neuropathic conditions. These findings can then be compared to clinical, behavioural and histological parameters to eventually provide a better understanding of DPN-related nerve lesions.

## Material and Methods

### Human nerve tissue for cross-platform validation

The study was approved by the local ethics committee of the University of Heidelberg (No. S-279/2018 & S-281/2018), and was performed in accordance with the Declaration of Helsinki 2013. All participants involved in this study gave written informed consent.

To compare MRN findings of diabetic nerve tissue between different platforms *in vivo* and *ex vivo*, we identified a 39-yo male patient who presented to our hospital for emergency amputation of his left leg due to an advanced diabetic foot syndrome with necrosis and non-healing wounds in the setting of severe diabetic neuropathy. The patient had a history of more than 20 years of T1D with end stage diabetic nephropathy that had been treated by a kidney transplant one year prior to admission. He showed a HbA_1C_ of 9.0% and an eGFR of 105.8 ml/min*1.73 m². His BMI was 24.8. Cardiovascular risk factors included hypertension and smoking. His freshly dissected sciatic nerve was directly taken from the operating room to the 9.4 T MR facility and immediately scanned. Five days post amputation, his opposite, non-amputated distal thigh was scanned under clinical conditions at 3 T.

For *ex vivo* comparison we identified a non-diabetic 74-yo male patient who presented to our hospital for emergency amputation of his left leg due to acute ischemia in the setting of stage IV peripheral artery disease and mild chronic kidney disease (G2A1) due to granulomatosis with polyangiitis (GPA). His history was further remarkable for pulmonary and ocular involvement of GPA, glaucoma and corneal ulcer. The patient showed a HbA_1C_ of 5.2% and an eGFR of 88.5 ml/min*1.73 m². No polyneuropathic symptoms were reported. For *in vivo* comparison, an age-matched healthy control subject (36-yo male, HbA_1C_ 5.0%, no polyneuropathic symptoms) underwent standard clinical MRN at 3 T.

### Animal model

All procedures were approved by the local Animal Care and Use Committee (Regierungspräsidium Karlsruhe, Germany; G295/15), and performed according to the guidelines of German animal welfare law. Diabetes was induced by administration of STZ via intraperitoneal injection (50 mg/kg body weight in 50 mM Sodium citrate; pH 4.5) on five consecutive days in ten-week old male and female C57BL/6 mice (Charles River Laboratories, Wilmington, MA, USA), whilst matched controls received sodium citrate (Table 1). Mice were maintained in the diabetic conditions (blood glucose >300 mg/dl) for 24 weeks by regular monitoring of blood glucose, sampled from the tail vain, and corrected by treatment with insulin^15^. This long period was selected to avoid artefacts introduced by the STZ treatment itself^16^. Glycated hemoglobin (HbA_1c_) was determined by cation-exchange chromatography on a PolyCAT A column^17^. Albumin-creatinine ratio was determined from 24 h urine collection and measured using a combined fluorometric and colorimetric microplate assay (Biovision, Milpitas, CA, USA), according to the manufacturer’s instructions.

**Table 1:** Basic clinical characteristics of the mouse study cohorts.

Phenotype	STZ	Control	*p-value*
Gender (m:f)	7:6	5:5	
Body weight (g)	21.9 ± 3.5	32.8 ± 5.3	*<0.0001*
Blood glucose (mg/dl)	418.2 ± 123.7	133 ± 18.6	*<0.0001*
HbA_1C_ (%)	9.5 ± 2.2	3.7 ± 0.4	*<0.0001*
Albumin-Creatinine Ratio (mg/g)	18.3 ± 9.4	6.9 ± 2.1	*<0.001*

### Quantitative sensory testing

Sensitivity to heat-induced pain was measured using an electronically controlled hot-plate analgesia meter (Columbus Instruments, Columbus, Ohio, USA) at 50 °C, as described previously^18^ and the nociceptive threshold was determined by the tail flick assay^15,19^. Additionally, the foot withdrawal latency was measured using the Hargreaves apparatus (Ugo Basile, Comerio, Italy), as described previously^16,20^.

### MR imaging & data acquisition

#### Human nerve tissue

Clinical *in vivo* MRN imaging was performed on a 3 T MR scanner (Magnetom Trio, Siemens, Erlangen, Germany). The MR protocol included the following sequences using a 15-channel transmit-receive knee coil (Siemens, Erlangen, Germany) positioned at distal thigh level, matching the position of the amputation site of the contralateral leg. Images were acquired in axial orientation to the long axis of the thigh:

1. High-resolution T2-w turbo-spin-echo (TSE) sequence: 2D sequence, TE: 55 ms, TR: 7000 ms, echo train length: 13, spectral fat saturation, Field-of-View (FoV): 160 ×160 mm², acquisition matrix: 512 ×333, number of slices: 41, slice thickness: 3.5 mm, number of averages: 2, acquisition time: 03 min 46 s.
2. DTI-EPI sequence: 2D sequence, number of diffusion gradients: 20, TE: 92.8 ms, TR: 4000 ms, spectral adiabatic inversion recovery (SPAIR) fat saturation, FoV: 160 ×160 mm², acquisition matrix: 128 ×128, number of slices: 18, slice thickness: 4 mm, b-values: 0/1000 s/mm², flip angle: 180°, number of averages: 1, acquisition time: 03 min 58 s.
3. Multi-Slice-Multi-Echo (MSME) sequence: 2D sequence, shortest TE and TE spacing: 10 ms, number of echoes: 12, spectral fat saturation, TR: 2400 ms, FoV: 160 ×160 mm², acquisition matrix: 192 ×169, number of slices: 11, slice thickness: 3.5 mm, number of averages: 1, acquisition time: 06 min 50 s.

For *ex vivo* imaging, freshly dissected sciatic nerves were immediately stored in Dulbecco’s Modified Eagle Medium/Nutrient Mixture F-12 (Thermo Fisher Inc., Waltham, MA, USA), put on ice and directly taken to the experimental MR unit. MR imaging was performed at room temperature on the same experimental 9.4 T system following the parameter settings as detailed below in section 2.3.2, except of sequence 2).

#### Mouse model

For MR imaging, animals were anesthetized with 3% isoflurane. Anesthesia was maintained with 1–2% isoflurane. Mice were placed on a heating pad in a supine position to keep the body temperature constant. Respiration was monitored externally using a breathing surface pad controlled by a custom-written LabView program (National Instruments Corporation, Austin, TX, USA).

MR imaging was performed on a 9.4 Tesla horizontal bore small animal NMR scanner (BioSpec 94/20 USR, Bruker BioSpin GmbH, Ettlingen, Germany) with a four-channel phased-array surface receiver coil, as described previously^21^. The MR protocol included the following sequence settings:

1. High-resolution T2-w Rapid Acquisition with Refocused Echoes (RARE) sequence with flip-back technique of the pelvis: 2D sequence, echo time (TE): 40 ms, repetition time (TR): 2500 ms, rare factor 4, spectral fat saturation, 47 µm x 47 µm in plane resolution, acquisition matrix: 512 ×212, number of slices: 15, slice thickness: 1 mm, number of averages: 2, acquisition time: 4 min 35 s.
2. High-resolution T2-w RARE sequence with flip-back technique of each proximal thigh: same parameters as in 1) except for TR: 4000 ms, acquisition matrix: 200 ×200, number of slices: 10, number of averages: 3, acquisition time: 10 min 17 s.
3. DTI-stimulated echo sequence of the pelvis: 2D sequence, number of diffusion gradient directions: 18 + 5 A0 images, b-values: 0/650 s/mm², gradient duration: 2.5 ms, gradient separation: 15.5 ms, TE: 18.1 ms, TR: 1200 ms, excitation pulse: 130°, spectral fat saturation, 100 µm x 128 µm in plane resolution, acquisition matrix: 120 ×50, number of slices: 17, slice thickness: 1.5 mm, number of averages: 1, acquisition time: 23 min 05 s.
4. MSME sequence of the pelvis: 2D sequence, shortest TE and TE spacing: 9.7 ms, number of echoes: 20, TR: 3200 ms, spectral fat saturation, 80 µm x 80 µm in plane resolution, acquisition matrix: 300 ×127, number of slices: 15, slice thickness: 1 mm, number of averages: 1, acquisition time: 7 min 12 s.

ADC and FA maps were calculated using Paravision 6.0 (Bruker BioSpin GmbH, Ettlingen, Germany). T2 and PD maps were calculated using a custom-written Matlab routine based on monoexponential fitting (R2015a, MathWorks Inc., Natwick, MA, USA)^22^. To enable a quantitative comparison of the signal intensity (SI) between different T2-w measurements, the human nerve SI was normalized to the SI of either the synovial fluid of the knee (*in vivo*) or the surrounding medium (*ex vivo*) according to T2w_norm_ = SI_nerve_/SI_fluid_. In the animal model, nerve SI was normalized to the SI of adjacent normal-appearing muscle according to T2w_norm_ = SI_nerve_/SI_muscle_.

### Histological examination

Following MRI, mice were killed using carbon dioxide and both sciatic nerves from three randomly picked STZ-diabetic and control mice were dissected and processed for analysis.

For light and electron microscopy, sciatic nerve specimens were cut to cubes of 3-5 mm length and fixed for at least 2 h at room temperature in 3% glutaraldehyde solution in 0.1 M cacodylate buffer pH 7.4, cut into pieces of ca. 1mm^3^, washed in buffer, postfixed for 1 h at 4° in 1% aqueous osmium tetroxide, rinsed in water, dehydrated through graded ethanol solutions, transferred into propylene oxide, and embedded in araldite resin^23^. Semithin and ultrathin sections were cut with an ultramicrotome (Reichert Ultracut E, Wien, Austria). Semithin sections of 0.9 µm were stained with toluidine blue for light microscopical examination (Hamamatsu NanoZoomer Digital Pathology, Hamamatsu Photonics, K.K., Japan) and digitized using NIS-Elements BR 3.00 Imaging Software (Nikon, Chiyoda, Japan). Selected areas of blocks were cut into 60-80 nm ultrathin sections, treated with uranyl acetate and lead citrate, and examined with an electron microscope JEM 1400 equipped with a 2 K TVIPS CCD Camera TemCam F216.

### Image evaluation and statistical analysis

MRN and histological images were exported and evaluation was performed in ImageJ Fiji^24^ using standard annotation and segmentation tools.

Human fascicular diabetic nerve lesions were identified on *in vivo* and *ex vivo* T2-w sequences and correspondingly segmented on images of the other MR contrasts. Likewise, inconspicuous nerve fascicles were identified and marked in the control cases.

For the quantitative morphometric analysis of myelinated fibres of the whole-mount, toluidine blue stained sciatic mouse nerves, axon density was defined as the absolute number of axons divided by the entire nerve cross sectional area; myelin density was defined as the absolute myelin area divided by the entire nerve cross sectional area and the average myelination was defined by absolute myelin area divided by the absolute number of myelinated axons within an entire nerve cross section.

For morphometric analysis of unmyelinated fibres, axon area, axon number and density and distribution of axon size were measured within randomly selected frames obtained from ultrathin-sections, each covering an area of 31 µm x 31 µm (n = 16 in control animals vs. n = 17 in STZ-animals). Ultrastructure of myelination was assessed visually in these image frames.

For further statistical analysis Prism Version 7.02 (GraphPad Software Inc., La Jolla, CA, USA), Microsoft Excel 2016 (Microsoft Inc., Redmond, WA, USA) and custom-written routines in MatLab (R2015a, MathWorks Inc., Natwick, MA, USA) were employed. Data are expressed as mean ± s.d. Group statistics were calculated using unpaired two-tailed Student’s t-test with Welch’s correction: *=p < 0.05, **p < 0.01, ***p < 0.001, ****p < 0.0001, n.s.=nonsignificant. Correlation tables were calculated using non-parametric Spearman’s r at a significance level of p < 0.05. Histogram comparison was carried out using two-tailed nonparametric Wilcoxon matched-pairs signed rank test at a significance level of p < 0.05.

## Results

### Long-term STZ-diabetic phenotype

**Figure 1:**
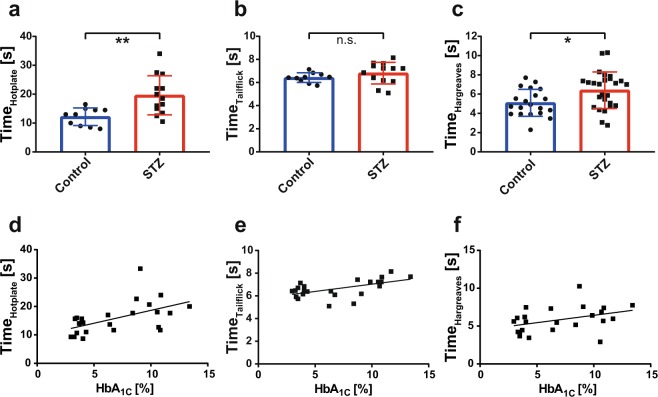
Behavioural phenotyping of STZ-induced diabetic mice. (**a**–**c**) Thermal nociception was tested measuring the behavioural latency [s] using the three different assays ‘Hotplate’, ‘Tailflick’ and ‘Hargreaves’. Significant differences between STZ-induced mice and controls were noted in the ‘Hotplate’ and ‘Hargreaves’. Group data provided as group mean ± s.d. *=p < 0.05, **=p < 0.01, n.s. = nonsignificant, unpaired two-sided t-test. N = 10 vs. 13 mice for ‘Hotplate’ and ‘Tailflick’ assays, n = 20 vs. 26 limbs for ‘Hargreaves’ assay. d-f) Significant correlation was noted for all of the behavioural assays and HbA_1c_ values (Hotplate: r = 0.59, p = 0.003; Tailflick: r = 0.59, p = 0.003; Hargreaves: r = 0.43, p = 0.04). Linear regression graphs shown (n = 23 mice).

STZ-diabetic mice showed a greatly reduced body weight, increased blood glucose level, increased HbA_1c_ and an increased Albumin-Creatinine ratio as compared to the control cohort (Table 1). STZ-diabetic mice exhibited a significant delay of the behavioural response in the hotplate and Hargreaves tests (Fig. 1a, c). Beside body weight, HbA_1c_ was found to be the only clinical parameter which significantly correlated to all three behavioural tests (Fig. 1d-f). These findings support the assumption of a robust hypoalgesic phenotype in mice with long-term STZ-induced diabetes.

### Ultra high field MRN of human diabetic nerve tissue

**Figure 2:**
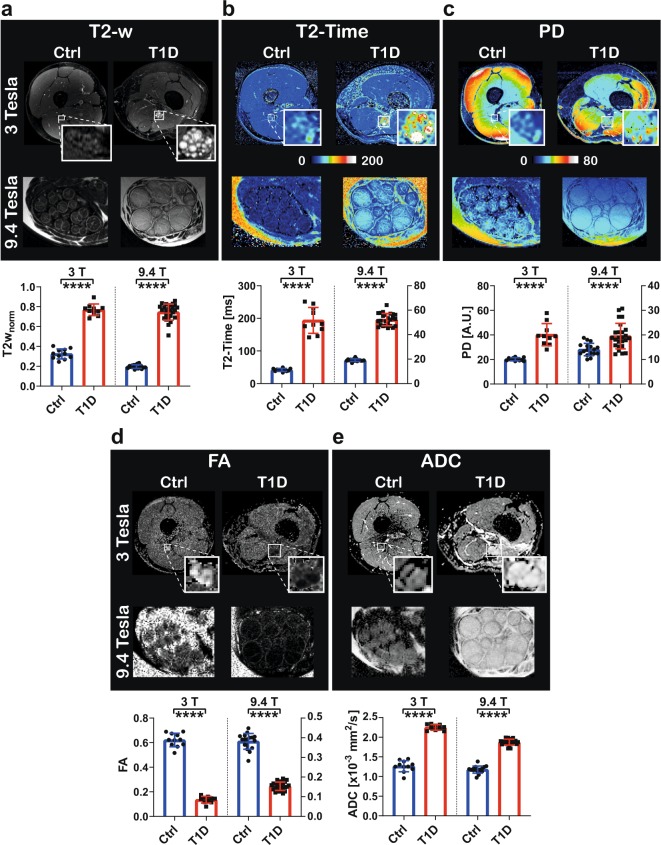
Cross-platform validation of multicontrast MRN at 3 and 9.4 T. (**a**–**e**) Illustration and corresponding quantitative findings of the five MR contrasts ‘T2w_norm_‘, ‘T2-Time’, ‘proton density (PD)’, ‘fractional anisotropy (FA)’ and ‘apparent diffusion coefficient (ADC)’. Each quadruplet of images shows a representative example MR-image of the control (Ctrl) and the type 1 diabetic (T1D) nerves at 3 and 9.4 T, respectively. A comparison of the respective quantitative fascicle group data is provided underneath, given as as group mean ± s.d. N = 10 (3 T) / 25 (9.4 T) vs. 11 (3 T) / 17 (9.4 T) fascicles, ****=p < 0.0001, unpaired two-sided t-test.

To allow for detailed visualization as well as morphological and quantitative evaluation of nerve tissue at a very high resolution, we developed an adapted multicontrast ultra high field MRN approach. We first intended to establish that tissue contrasts obtained at 9.4 T magnetic field strength would indeed reflect the same findings as acquisition under clinical conditions *in vivo*. We therefore performed a cross-platform validation experiment in which we compared *in vivo* and *ex vivo* nerve tissue from a T1D patient with the findings of non-diabetic nerves (Fig. 2). At 3 T, T2w_norm_ was 0.77 ± 0.06 vs. 0.32 ± 0.05 (p < 0.0001, Fig. 2a), T2-time was 193.6 ± 39.7 vs. 41.9 ± 4.4 ms (p < 0.0001, Fig. 2b), PD was 40.18 ± 9.04 vs 20.21 ± 1.29 (p < 0.0001, Fig. 2c), FA was 0.14 ± 0.03 vs. 0.62 ± 0.05 (p < 0.0001, Fig. 2d) and ADC was 2.25 ± 0.07 vs. 1.26 ± 0.14 *10^-^³mm²/s (p < 0.0001, Fig. 2e). At 9.4 T, T2w_norm_ was 0.75 ± 0.08 vs. 0.20 ± 0.02 (p < 0.0001, Fig. 2a); T2-time was 52.3 ± 5.01 vs. 19.2 ± 1.05 ms (p < 0.0001, Fig. 2b), PD was 19.51 ± 5.21 vs 14.05 ± 2.54 (p < 0.0001, Fig. 2c), FA was 0.15 ± 0.02 vs. 0.38 ± 0.04 (p < 0.0001, Fig. 2d) and ADC was 1.88 ± 0.07 vs. 1.18 ± 0.09 *10^-^³mm²/s (p < 0.0001, Fig. 2e).

These findings show that for each individual MR parameter considered, the same expected tissue contrast changes can be visualized using the experimental MRN protocol at 9.4 T as in the clinical *in vivo* setting at 3 T. We therefore conclude that our multicontrast approach at ultra high field strength can readily be used to detect type 1 DPN nerve lesions.

### Ultra high field MRN in the STZ-diabetic mouse model

**Figure 3:**
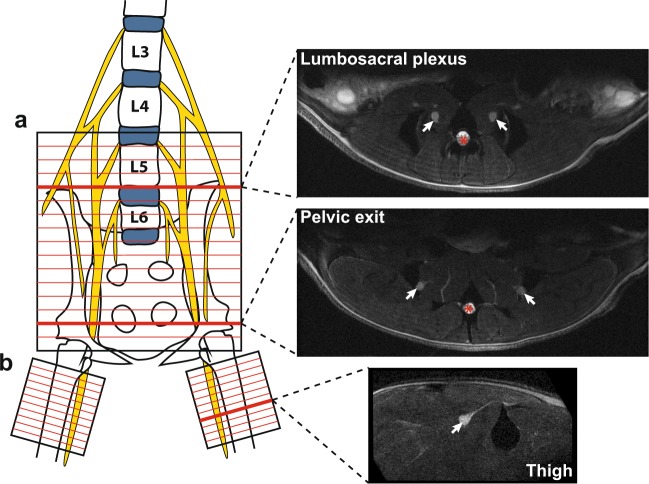
Imaging rationale of ultra high field MRN in experimental DPN. Schematic of the lumbosacral plexus with indication of imaging regions. The upper two images show normal morphological T2-w images of both sciatic nerves (white arrows) within the multicontrast imaging region of the lumbosacral plexus and at the pelvic exit in an axial orientation (**a**). Lower image depicting a normal axial T2-w image plane of the sciatic nerve along the proximal thigh (**b**), also indicated by a white arrow. For better orientation, the spinal canal in the image center, surrounded by the hypointense lumbar vertebral column, is indicated by red asterisks.

Applying this technique to the mouse model, our strategy consisted of a proximal multicontrast MRN protocol which was complemented by morphological T2-w MRN of both sciatic nerves at thigh level (Fig. 3, sciatic nerve indicated by white arrows).

**Figure 4:**
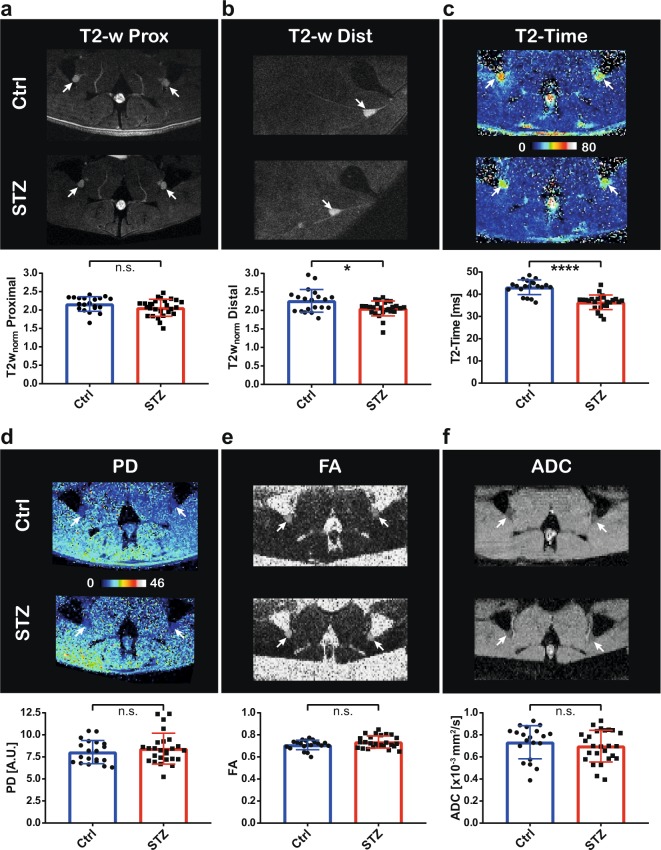
Multicontrast MRN in STZ-diabetic mice at ultra high field strength. (**a**–**f**) Illustration and corresponding quantitative findings of the six MR contrasts ‘T2w_norm_‘ at proximal (i.e. pelvic level) and distal (i.e. thigh level) positions, ‘T2-time’, ‘proton density (PD)’, ‘fractional anisotropy (FA)’ and ‘apparent diffusion coefficient (ADC)’. Each pair of images shows a representative image example of the control (Ctrl) and STZ group, respectively. A comparison of the respective quantitative group data is provided underneath, given as group mean ± s.d. N = 20 vs. 26 nerves. *p < 0.05, ****p < 0.0001, n.s. = nonsignificant, unpaired two-sided t-test. White arrows indicating sciatic nerves.

Other than expected, there was a significant shortening of the T2-time in the sciatic nerve and a significant decrease in T2w_norm_ at the thigh level in STZ-diabetic mice. No significant difference was noted in the T2w_norm_ of the proximal position at the pelvic exit between STZ-diabetic and control mice (Fig. 4a-c). However, neither focal nerve signal nor morphological alterations were identified in the STZ-diabetic mice that would correspond to the pathognomonic lesions earlier reported in patients with DPN and also observed in our validation experiment on human T1D nerve tissue. Quantitative analysis of PD and the water diffusion parameters FA and ADC did not yield any significant differences (Fig. 4d-f).

**Figure 5:**
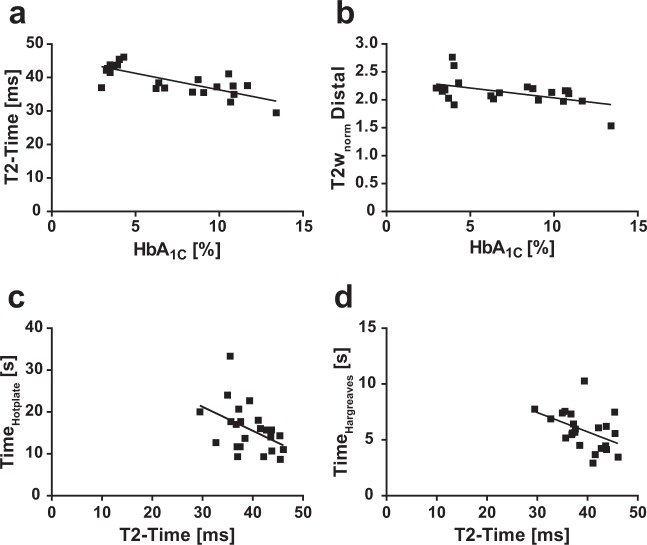
Correlation analysis of MRN parameters with clinical and behavioural variables. Significant correlation was noted for the MRN parameters (**a**) ‘T2-time’ (r = −0.62, p = 0.002) and (**b**) ‘T2w_norm_ Distal’ (r = −0.51, p = 0.014) with the HbA_1c_ value and for T2-time with the behavioural assays (**c**) ‘Hotplate’ (r = −0.49, p = 0.018) and (**d**) ‘Hargreaves’ (r = −0.49, p = 0.017). Corresponding linear regression graphs shown (n = 23 mice).

Correlation analysis of MRN parameters with clinical and behavioral parameters showed that HbA_1c_, as the most robust clinical marker, significantly correlated to T2-time (r = -0.62, p = 0.002) and to T2w_norm_ at distal level (r = −0.51, p = 0.02) (Fig. 5a, b), whilst all other MRN parameters failed to do so. It was subsequently found that T2-time was the only MRN parameter which correlated significantly with either the hotplate (r = -0.49, p = 0.02) or Hargreaves assay (r = -0.49, p = 0.02) (Fig. 5c, d), but not the tail-flick.

### Histological and ultrastructural nerve analysis

**Figure 6:**
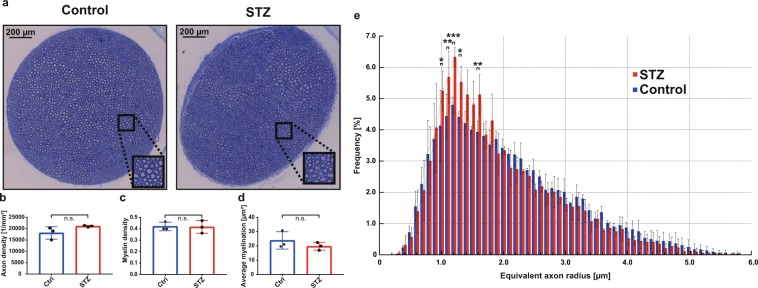
Light microscopic analysis of the sciatic nerves from STZ-diabetic mice. (**a**) Gross anatomical findings of Toluidine blue stained semithin sciatic nerve sections from control and STZ animals using light microscopy (LM). Inset shows a representative region in x2-magnification. (**b**–**d**) Quantitative findings of the control (Ctrl) and the STZ groups (n = 3 each) comparing the three parameters b) ‘axon density’, (**c**) ‘myelin density’ (i.e. the myelin fraction within the nerve cross-sectional area (CSA)) and (**d**) ‘average myelination’ (i.e. myelin area per axon); n.s. = nonsignificant, unpaired two-sided t-test. (**e**) Histogram plot showing the frequency distribution of the equivalent axon radius within the population of all myelinated axons inside the nerve CSA. Each bar indicates group mean ± s.d. (n = 3 each). *p < 0.05, **p < 0.01, ***p < 0.001, unpaired two-sided t-test.

Quantitative evaluation of whole-mount, toluidine blue stained semithin-sections of sciatic nerves (Fig. 6a) from control and STZ-diabetic mice showed no significant differences in either overall myelinated axon density (18087 ± 2725 vs. 21069 ± 369.5 per mm²; p = 0.20; Fig. 6b) or myelin density (0.42 ± 0.021 vs. 0.416 ± 0.055 µm²; p = 0.91; Fig. 6c). With respect to average myelination, there was a 17% reduction in the sciatic nerves from the STZ-diabetic mice; however, this difference was nonsignificant (23.9 ± 6.08 vs. 19.8 ± 2.75 µm²; p = 0.37; Fig. 6d). Exact frequency distribution analysis of all myelinated axons, however, showed a left shift towards smaller fibers in STZ-diabetic mice (Fig. 6e) with a significant increase of small radius axons between 1-2 µm when compared to the control cohort. This shift could also be confirmed by pairwise comparison of the mean axon size distribution (p < 0.01, Wilcoxon-signed-rank test). In the overall group data, this leads to a 22% decrease of the mean equivalent axon radius (2.36 ± 0.56 vs. 1.84 ± 0.76 µm; p = 0.49).

**Figure 7:**
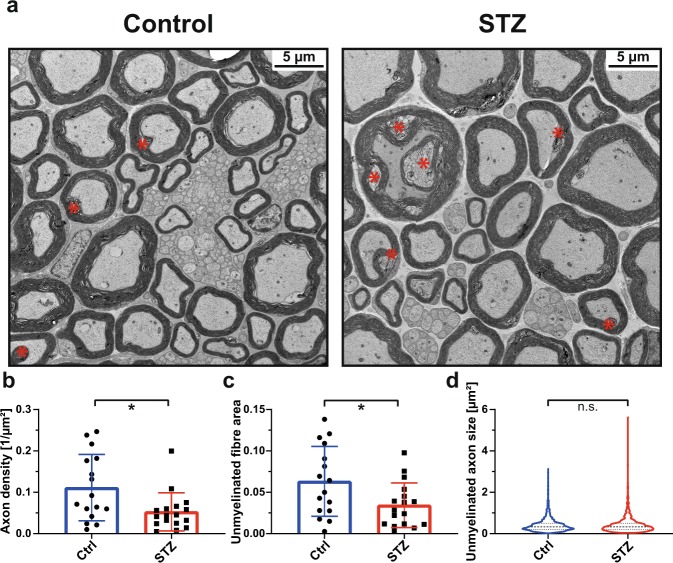
Ultrastructural analysis of the sciatic nerves from STZ-diabetic mice. (**a**) Representative ultrastructural findings from control and STZ animals using electron microscopy (EM). Red asterisks indicate myelin infoldings. (**b**–**d**) Quantitative findings of the control (Ctrl) and the STZ groups comparing the three parameters (**b**) Unmyelinated ‘axon density’ (per image frame, n = 16 vs. 17 frames), (**c**) ‘Unmyelinated fibre area’ (i.e. the unmyelinated fibre fraction per image frame, n = 16 vs. 17 frames) and d) ‘Unmyelinated axon size’ (i.e. all unmyelinated fibres within the image frames considered, median and quartiles indicated, n = 1646 (Ctrl) vs. 878 (STZ)); *=p < 0.05, n.s. = nonsignificant, unpaired two-sided t-test.

Despite the presence of a number of artifacts in the respective images, electron microscopy (EM) showed an increase in myelin fiber abnormalities such as infoldings (4147 ± 1489 vs. 1990 ± 1315 per mm²; p < 0.01; Fig. 7a) in sciatic nerves from STZ-diabetic mice, which may be consistent with pathological changes previously reported for peripheral neuropathy. In contrast, alterations in myelin compaction were not found to occur more often in STZ-diabetic mice (2483 ± 1136 vs. 1842 ± 1334 per mm²; p = 0.15).

Furthermore, quantitative EM morphometry showed a significant decrease of unmyelinated axon density (0.053 ± 0.046 vs. 0.111 ± 0.08; p = 0.02) and unmyelinated fiber area (0.034 ± 0.027 vs. 0.063 ± 0.042; p = 0.03) in sciatic nerves of STZ-diabetic mice compared to the control cohort (Fig. 7b, c). While there was a nonsignificant trend towards an increased proportion of small-sized unmyelinated axons <0.2 µm² (0.32 ± 0.15 vs. 0.24 ± 0.07; p = 0.08), there was a significant smaller proportion of medium-sized unmyelinated axons between 0.2 µm² and 0.8 µm² (0.59 ± 0.12 vs. 0.66 ± 0.08; p = 0.04) in the sciatic nerves of STZ-diabetic mice. The overall size distribution of all unmyelinated axons, however, appeared to be very similar as evidenced by the violin plot (Fig. 7d, median 0.33 vs 0.33, 25^th^ percentile 0.198 vs. 0.209, 75^th^ percentile 0.503 vs. 0.504, n = 878 vs. 1646; p = 0.54).

## Discussion

MRN has proven to be a powerful and innovative tool readily depicting peripheral nerve lesions in human DPN with proximal predominance^1–3^. In addition, the lesion load has been found to correspond closely to the clinical severity of DPN^1–3^ and seems to mirror earlier pathological findings of multifocal fiber loss along the nerve^12,25^. The description of such nerve signal alterations has been broadened by the use of DTI^5,6^ and T2-relaxometry^2,6^ assigning a well-defined “multicontrast MR-signature” to these characteristic DPN lesions.

Rodents are frequently used as model organisms in the study of DPN to identify possible therapeutic targets. While *in vivo* assessment of clinical phenotype or physiological functions is often difficult, objective tools to monitor morphological and functional changes over time appear highly desirable.

Here, we have introduced a novel multicontrast MRN approach at ultra high field strength for the high-resolution *in vivo* study of the PNS in mice, based upon human clinical MRN strategies.

To validate this experimental approach and confirm that results acquired at 9.4 T in an experimental setting are comparable to human clinical findings *in vivo*, we have first come up with an elaborate cross-platform strategy involving the parallel comparison of *ex vivo* and *in vivo* sciatic nerve tissue from a patient with long standing T1D and severe dPNP to non-diabetic controls. This could show that our experimental ultra high field MRN approach was indeed able to pick up the same typical signal features of type 1 DPN as conventional *in vivo* MRN in patients. One important limitation to this approach, however, is the validation in only a single case-control comparison with serious co-morbidities and accompanying medication which could have introduced a considerable bias. On the other hand, the observed extensive MRN phenotype in T1D with stereotypical signal alterations matched closely previously reported cases of the same condition^1–3,6^ so there is no reason for us to believe these findings to be artifacts of other etiology. Furthermore, it seems very difficult to identify patients, especially T1D, with such a severe dPNP warranting a major leg amputation without any other substantial diabetic late complications or co-morbidities to completely exclude potential contributions from other physiological systems. To gain *ex vivo* access to major peripheral nerves, it seems nearly impossible, unfortunately, to get around this shortcoming.

We applied our experimental MRN strategy to the STZ diabetic mouse, one of the most frequently studied DPN animal models. Surprisingly, MRN findings in these mice were strikingly different from the characteristic MR-signature observed in patients with type 1 DPN at 3 and 9.4 T. Although exhibiting typical clinical and behavioural phenotype featuring thermal hypoalgesia, there were no classic MR-morphological focal lesions or nerve enlargement^1,4^ nor the expected quantitative changes, such as an increase in T2-w SI^1^, proton density^2^, a decrease in FA or an increase in ADC^5,6^. Instead, a marked drop of T2-time and a corresponding reduction in general T2-w signal of the nerve were identified which is contrary to prior reports on T2-time in patients with DPN^2,5^. In fact, T2-w hyperintensity was the first reported major hallmark of human DPN nerve lesions^1,4^.

As the exact nature of such lesions in humans still remains unclear, it would be unwise to speculate as to the reasons for their absence from the mouse but the difference could be due to subtler nerve pathology and the lack of gross changes in axonal number – which is a known observation from earlier studies in STZ-diabetic rodents^14,26^. Indeed, the alterations observed on the light-microscopic and ultrastructural level in our study could reproduce earlier findings in STZ-diabetic rodents which are thought to be linked to experimental DPN: Among those, an overall left-shift of myelinated fibres, in particular, appears to be a most consistent finding^26–28^. Although to a lesser extent, this does also seem to be the case for unmyelinated axons with a significant reduction in overall unmyelinated fibre area^26^. A reduction in unmyelinated axon density as we found has been reported less frequently^29^ but may – at our relatively late observational time point – reflect a later finding of well-known dying back degeneration of C-fiber terminals^8,30–33^. Moreover, myelin irregularities could also be found which is consistent with earlier reports^14,34^.

As proposed in previous studies^26^, the observed phenotype and structural morphotype of the STZ-diabetic animals may reflect early diabetic neuropathy in humans given the absence of gross nerve pathology compared to human DPN^12^. Therefore, the shortened T2-time observed in STZ-diabetic mice in our study could be explained by the altered axonal composition of the nerve due to the left-shift of the size distribution: An increase of fiber numbers with smaller fiber radii and decreased average myelination would lead to a greater surface area of cellular membranes within the nerve, which is believed to result in a T2 decrease^35^. Also, such changes are likely to have an impact on tissue susceptibility additionally contributing to a shortening of the T2-time^36^.

Our findings could therefore represent an early indicator of structural re-arrangement within the nerve, potentially preceding the MR-signature of late-stage diabetic neuropathy. Translational and confirmatory studies are therefore required to determine if these structural changes are indeed responsible for the MR-parameter changes observed and how they may be related to the neuropathic phenotype.

It has previously been reported that injured peripheral nerves within an experimental context have an increased T2-w SI and prolonged T2 values, followed by gradual recovery and normalization of T2 signal over time^37–39^. In a study with STZ-diabetic rats, similar findings have also been reported^40^. This, however, contrasts with the findings of our study, which observed an unexpected decrease of T2-time in the STZ mice. This might be due to differences between rat and mouse, particularly, with regard to STZ treatment. In the rat study by Wang et al., a single 50 mg/g dose of STZ was used to induce persistent hyperglycemia and an increase in tactile allodynia as assessed by von Frey filament seven weeks post-induction; but this duration may be too short for the development of a true DPN morphotype within the peripheral nervous system and STZ-related artifacts are still expected to be present^16^, potentially leading to an endoneurial oedema that can be picked up by an increase in T2-w SI. In contrast, the mice in our study received five consecutive injections of STZ at the same dose and significant differences in thermal hyperalgesia were observed after 24 weeks, a widely accepted paradigm of experimental DPN. Under these conditions, we did not find any sign of endoneurial oedema. Such differences highlight the need for standardized models in the future to achieve an effective comparison even across species. In addition to the MR measurements, complementary parameters, such as IENFD, should be performed to assess the relationship between dermal fiber loss and the integrate of the sciatic nerve.

In conclusion, we have implemented a novel multicontrast ultra high field MRN approach for the *in vivo* study of major peripheral nerve segments in mice. Within the context of the standard STZ model, there was no evidence for the presence of characteristic lesions within the proximal sciatic nerve as has previously been reported in patients with DPN. However, an unexpected, distinct MR-signature was observed that may represent an indicator of early structural re-arrangement within the nerve in the context of DPN. The capacity of our MRN approach for non-invasive assessment of proximal nerve structure and function within a given mouse model provides a powerful tool for direct translational comparison to human disease hallmarks not only in diabetes but also in other peripheral neuropathic conditions.

## Data Availability

Data are available from the corresponding author upon reasonable request.
